# Identifying protein complex by integrating characteristic of core-attachment into dynamic PPI network

**DOI:** 10.1371/journal.pone.0186134

**Published:** 2017-10-18

**Authors:** Xianjun Shen, Li Yi, Xingpeng Jiang, Tingting He, Jincai Yang, Wei Xie, Po Hu, Xiaohua Hu

**Affiliations:** 1 School of Computer, Central China Normal University, Wuhan, China; 2 Letv Cloud Computing Co. Ltd, Beijing, China; 3 College of Computing and Informatics, Drexel University, Philadelphia, United States of America; National Centre For Cell Science, INDIA

## Abstract

How to identify protein complex is an important and challenging task in proteomics. It would make great contribution to our knowledge of molecular mechanism in cell life activities. However, the inherent organization and dynamic characteristic of cell system have rarely been incorporated into the existing algorithms for detecting protein complexes because of the limitation of protein-protein interaction (PPI) data produced by high throughput techniques. The availability of time course gene expression profile enables us to uncover the dynamics of molecular networks and improve the detection of protein complexes. In order to achieve this goal, this paper proposes a novel algorithm DCA (Dynamic Core-Attachment). It detects protein-complex core comprising of continually expressed and highly connected proteins in dynamic PPI network, and then the protein complex is formed by including the attachments with high adhesion into the core. The integration of core-attachment feature into the dynamic PPI network is responsible for the superiority of our algorithm. DCA has been applied on two different yeast dynamic PPI networks and the experimental results show that it performs significantly better than the state-of-the-art techniques in terms of prediction accuracy, hF-measure and statistical significance in biology. In addition, the identified complexes with strong biological significance provide potential candidate complexes for biologists to validate.

## Introduction

Cellular functions are completed by protein complex formed by multiple proteins aggregating together, rather than by individual protein. Identifying protein complex has significant implications in revealing the important principle of protein organization within cell [1, 2]. Protein complexes can help us to predict the functions of protein [3]. Accumulated evidences suggest that protein complexes are involved in many disease mechanisms [4]. Tracking the protein complexes could reveal important insights into modular mechanisms and improve our understanding on the disease pathways [5].

In proteomics, large-scale protein-protein interaction (PPI) data have being produced along with high-throughput techniques such as yeast two-hybrid (Y2H) [6] and affinity purification [7]. Typically, PPI data are abstracted to a complex network model in which protein is regarded as node while interaction as edge. Such network is characteristic of modular structure and prompts the emergence of many computational approaches for detecting protein complexes.

Most of current methods are based on solely network clustering[8–10] or integrated with multiple biological data[11–16]. For example, Palla et al. proposed CPM (Clique Percolation Method) algorithm to detect overlapping dense groups of nodes as protein complexes by continuously merging maximal connected sub graphs containing *k* vertexes in PPI networks[17]. Review articles [1, 2, 18] provide insight into the contributions of the areas, which have significant meanings to reveal the important principles of protein organizations within cells.

We know that protein complex consists of highly connected proteins, but it is much more than that. Literature [19] indicates that protein complex is characteristic of core-attachment structure, which has given rise to many protein complex identifying algorithms based on such theoretical principle. For instance, COACH [20], CoreAttach [21] and PCD-GED [22] approaches. But they often neglect the inherent time sequential feature in cell life activities. Cellular systems are highly dynamic and responsive to the stimulus from external environment [23]. Han JD et al. has proved the dynamically organized modularity in yeast PPI network [24]. Thus it has important implications in making a transition from the analyzing of static PPI networks to dynamic networks[25].

In this paper, we propose a new algorithm, called DCA (Dynamic Core-Attachment), to identify protein complexes by integrating their inherent organizations into dynamic PPI network. Protein- complex cores are formed by continually expressed and highly connected proteins. We subsequently generate protein complexes by appending attachments into the protein-complex cores. The integration of core-attachment feature into the time-evolving PPI network is responsible for the superiority of our algorithm. Experimental results using two PPI data sets of *Saccharomyces cerevisiae* show that our DCA method outperforms existing computational methods in terms of prediction accuracy, hF-measure and statistical significance in biology.

## Materials and methods

To capture the dynamics of protein complex, time course gene expression data are integrated into the original static PPI network and generate the dynamic PPI network with three sigma method[26]. In brief it contains two steps. Firstly, for each gene at a time point, it is considered to be active only if its expression value is greater than a given threshold which is calculated based on three sigma principle. Secondly, the active proteins at this time point and their connections in the static network constitute a sub-network. As a result, all the time series sub-networks behave as a dynamic network. Please refer to the literature [26] for more detail.

Our DCA algorithm operates in four phases based on the dynamic network. DCA first identifies protein-complex cores and then applies an outward growing strategy to produce protein complexes by including attachments into the protein-complex cores. We will first briefly introduce some basic terminologies and then describe in detail our proposed method for protein complex detection.

### Preliminaries

Proteins in complex core that playing a central role are characteristic of highly connected, sharing the functions of the same classification and relatively stable, which means that they have a relatively long duration for activity. Based on such an assumption, for one thing their edges own higher edge clustering coefficient (referred as *ECC*, as shown in Eq (1)), for another the stability of protein activity (referred as *AT*) here is defined as the time span between the starting and ending time point of its active state. For example, suppose that one protein’s activity starts from time point 6 and it becomes inactive at time point 9, then its active time span (*AT*) of course is 3. To characterize effectively those biological nature of protein complex, we weight the PPI network by combining *ECC* and *AT* as shown in Eq (2):

$$ECC_{ij}=\frac{Z_{ij}}{\min(k_{i}-1,k_{j}-1)}$$ (1)

$$W_{v}=\alpha *\sum_{k\in N_{v}}ECC_{vk}+(1-\alpha )*AT$$ (2)

In Eq (1), *Z*_*ij*_ represents the number of common neighbors of the two interacting proteins *i* and *j*, while min(*k*_*i*_-1, *k*_*j*_*-*1) equals to the theoretical maximum number of triangles containing the two nodes. *ECC* ranges from 0 to 1 and the greater value shows the closer relationship among the nodes and their neighbors. In Eq (2), *N*_*v*_ contains the neighbors of node *v* and *AT* ranges from 0 to 1 after normalization. *α* controls the contribution proportion of *ECC* against *AT*. They are complementary and consistent with each other. First, due to the false negatives of protein interaction data, some of the interactions in protein-complex core will gain lower *ECC*, so it is reasonable to increase the weight with greater *AT*. Instead, some interactions outsize protein-complex core will gain higher weight because of the false positives of interactions, then it is also reasonable to decrease the weight with lower *AT*. Second, the greater the value of either *ECC* or *AT*, the greater the likelihood that they participate in central biological functions in protein-complex core.

As for the attachments of protein-complex, they participate in different protein complexes playing a variety of functions as a supporting role. Nevertheless, as a part of a whole protein complex, they still have relatively closer relationship with the complex core. We define this relationship as *adhesion* shown in Eq (3):

$$Adh_{s\_Core}=\sum_{v\in N_{S}\cap Core}ECC_{sv}$$ (3)

Where *s* is a neighbor of *Core*. *Adh*_*s_Core*_ describes the closeness between a protein-complex core and its neighbors, so we use it to measure the likelihood of that whether a protein should be include into a core as its attachment.

### DCA algorithm

**Fig 1:**
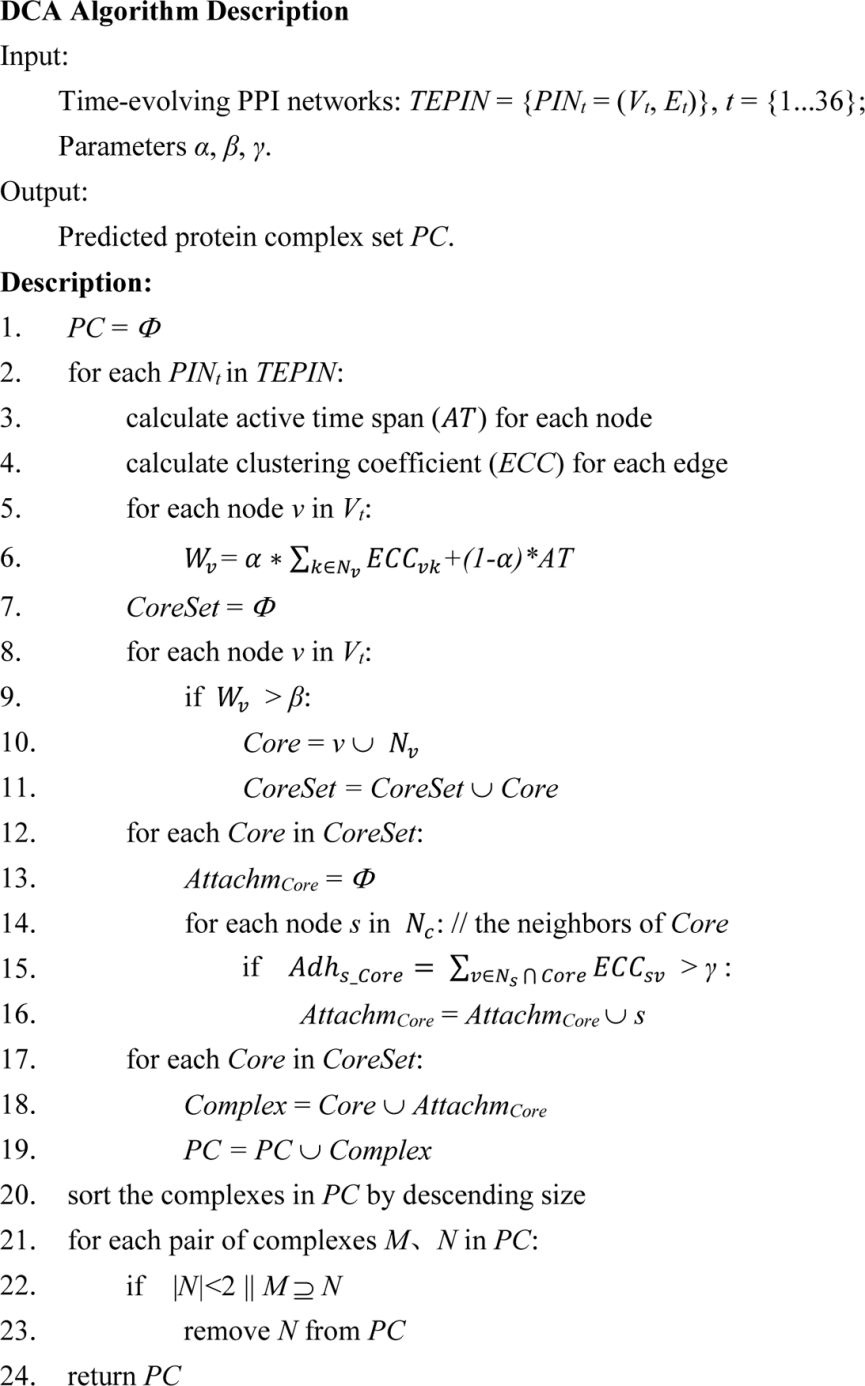
DCA algorithm description.

As shown in Fig 1, based on each snapshot of the dynamic PPI network, DCA algorithm firstly calculate the weight for each protein node according to its stability of activity and edge clustering coefficient (lines 3~6); Secondly, the nodes with weight greater than *β* are separately consolidated with their neighbors to form the protein-complex cores (lines 7~11). Thirdly, for each protein-complex core we select reliable attachments cooperating with it to form a protein complex (lines 12~19). Due to the periodic properties of gene expression data, the identified protein complexes contain large number of approximate ones. The last step is the redundancy-filtering procedure (lines 20~23). The computational complexity of DCA algorithm is *O*(*N*^2^) under given parameters of *α*, *β* and *γ*, where *N* is the number of nodes in network.

Our DCA algorithm operates on time-evolving PPI network and takes its time sequential feature into account. Besides, the predicted protein complexes may overlap with each other since the attachments typically participate multiple protein complexes to carry out specific biological functions.

### Experimental data

In order to verify the validity of the proposed DCA algorithm, we use two PPI networks of yeast: DIP [27] (Version of 20101010) and Krogan_extended [28] data sets. After filtering a small number of proteins that do not express the spectrum, the former contains 24278 interactions among 4969 proteins while the latter includes 12399 interactions among 3153 proteins. Gene expression data over three successive metabolic cycles are available from GEO (Gene Expression Omnibus) with accession number GSE3431[29]. This dataset includes the expression profiles of 9335 probes under 36 different time points. It is used to construct dynamic PPI network by integrating into the static PPI network. The known protein complex set containing 349 complexes after removing the one that is not covered by the PPI network is derived from CYC2008[30], which is widely used as a reference set of protein complexes to evaluate protein complex prediction and allows precise standardized functional descriptions of genes.

### Metrics for evaluating identified protein complexes

Three evaluating metrics, namely F-measure, GO enrichment analysis and hF-measure are used in this paper to test the performance of DCA algorithm.

Overlapping Score (*OS*)[12] Eq (4) is usually used to assess the match score between a predicted protein complex *pc* and a known protein complex *kc*:

$$OS(pc,kc)=\frac{{\left|pc\cap kc\right|}^{2}}{\left|pc\right|\times \left|kc\right|}$$ (4)

Where *pc∩kc* represents the number of the proteins involved in both complexes *pc* and *kc*; |*pc*| and |*kc*| represent the number of proteins involved in complex *pc* and complex *kc* respectively. Two protein complexes are considered to be matched if their overlapping score is greater than or equal to a given threshold, which is set to 0.2, the same as many other researches[12]. Particularly, *OS*(*pc*,*kc*) = 1 indicates that the two complexes *pc* and *kc* match perfectly.

The predicted protein complex sets identified by various algorithms are separately compared against the known protein complex set, by which we can obtain the performance of algorithms on Sensitivity (*Sn*) and Specificity (*Sp*). They are typically employed to evaluate the identification of protein complexes. Let true positives (*TP*) denote the number of predicted protein complexes that match with known complexes, false positives (*FP*) denote the number of unmatched ones, and false negatives (*FN*) denote the number of known protein complexes which match with none of the predicted ones, then *Sn* and *Sp* can be defined as Eq (5) and Eq (6), respectively. The harmonic mean of *Sn* and *Sp*, also known as *F-measure* Eq (7), is often used to assess the overall accuracy of various methods[12].

Sn=TP/(TP+FN) (5)

Sp=TP/(TP+FP) (6)

$$F-measure=\frac{2\times Sn\times Sp}{Sn+Sp}$$ (7)

Larger *Sn* to some extent indicates that more known protein complexes could be recognized, while higher *Sp* shows that higher percentage of predicted protein complexes match with known protein complexes.

To evaluate the statistical significance of the identified protein complexes, many researchers annotate their main biological functions by using p-value formulated as Eq (8) [26]. Given a predicted protein complex containing *C* proteins, p-value calculates the probability of observing *k* or more proteins from the complex by chance in a biological function shared by *F* proteins from a total genome size of *N* proteins:

$$p-value=1-\sum_{i=0}^{k-1}\frac{\left(\begin{array}{c} F\\ i \end{array}\right)\left(\begin{array}{c} N-F\\ C-i \end{array}\right)}{\left(\begin{array}{c} N\\ C \end{array}\right)}$$ (8)

The lower the p-value is, the stronger biological significance the complex possesses, while the complex with p-value greater than 0.01 is deemed to be meaningless at all. Generally speaking, the larger protein complexes possess the smaller p-values.

HF-measure is a measurement to evaluate clusters more finely and distinctly[31]. It uses functional annotation information in the GO database to measure the similarity between components in protein complexes. There are two versions of this metric—the one is topology-free measurement hF-measure^Tf^, the other is topology-based measurement hF-measure^Tb^. Unlike F-measure, the new measurements of hF-measure^Tf^ and hF-measure^Tb^ can discriminate between different types of errors.

## Results and discussion

For the gene expression data including 36 time points used in this paper, averagely there are 1043 (SD = 240) active proteins at each time point. By mapping them into the static PPI networks, the number distribution of proteins and interactions in sub-networks is shown in Table 1.

**Table 1:** The number distribution of proteins and interactions in the sub-networks of dynamic network.

Data set	Proteins	Interactions
DIP	Ave = 599, SD = 223	Ave = 898, SD = 668
Krogan_extended	Ave = 298, SD = 166	Ave = 427, SD = 530

Using two data sets, DIP and Krogan_extended, we have applied our DCA algorithm on two yeast dynamic PPI networks constructed with three sigma method[26] to perform comprehensive comparisons among various existing competing algorithms including DPC[32], TS-OCD[33], CAMSE[10], ClusterONE[9], SPICI[34], COACH[20], CoreAttach[21], CPM[35] and MCODE[36]. For all these methods, the optimal parameters are set to default empirical values, while in DCA we recommend *α* = 0.60, *β* = 0.55, *γ* = 1.4. Table 2 shows the basic information of predictions by various methods on the two dynamic PPI networks. On DIP data, DCA predicted 885 complexes with average size of 8.2, of which 515 match 118 real complexes; On Krogan_extended data, it predicted 818 complexes with average size of 8.7, of which 558 match 90 real complexes.

**Table 2:** The basic results of various algorithms on two dynamic networks.

	DIP					Krogan_extended				
Algorithms	#AS	#MS	#MPC	#PC	#MKC	#AS	#MS	#MPC	#PC	#MKC
DCA	8.2	47	515	885	118	8.7	35	558	818	90
DPC	-	-	-	766^[^^32^^]^	-	-	-	-	-	-
TS-OCD	5.1	25	279	843	162	5.6	21	187	314	114
CAMSE	4.3	22	881	2433	215	5.4	25	627	1185	158
ClusterONE	3.8	20	564	1690	213	4.5	20	526	1309	179
CoreAttach	3.3	30	652	2935	282	3.3	26	456	1402	213
CPM	7.2	345	249	531	125	7.6	349	200	300	83
MCODE	6.2	43	147	309	82	5.8	51	192	272	76
SPICI	4.2	25	551	2089	197	4.3	21	399	1011	155
COACH	5.6	26	361	899	150	6.3	27	286	482	101

#AS: the average size of predicted protein complexes; #MS: the maximum size of predicted protein complexes; #MPC: the number of predicted protein complexes matched by known protein complexes; #PC: the total number of predicted protein complexes; #MKC: the number of known complexes matched by predicted protein complexes

### Comparative sensitivity and specificity

**Fig 2:**
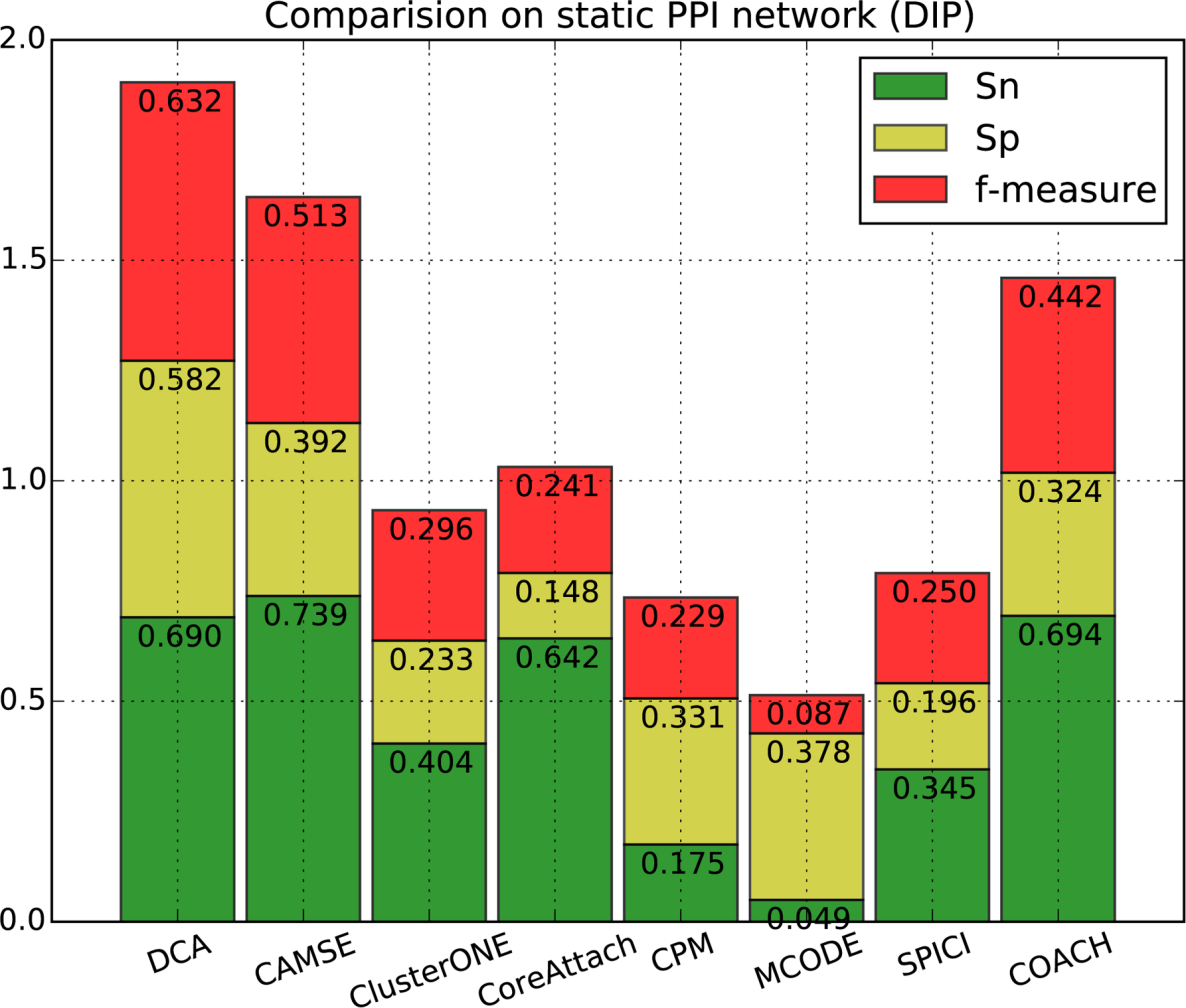
Performance comparison of DCA against other algorithms on static DIP network.

**Fig 3:**
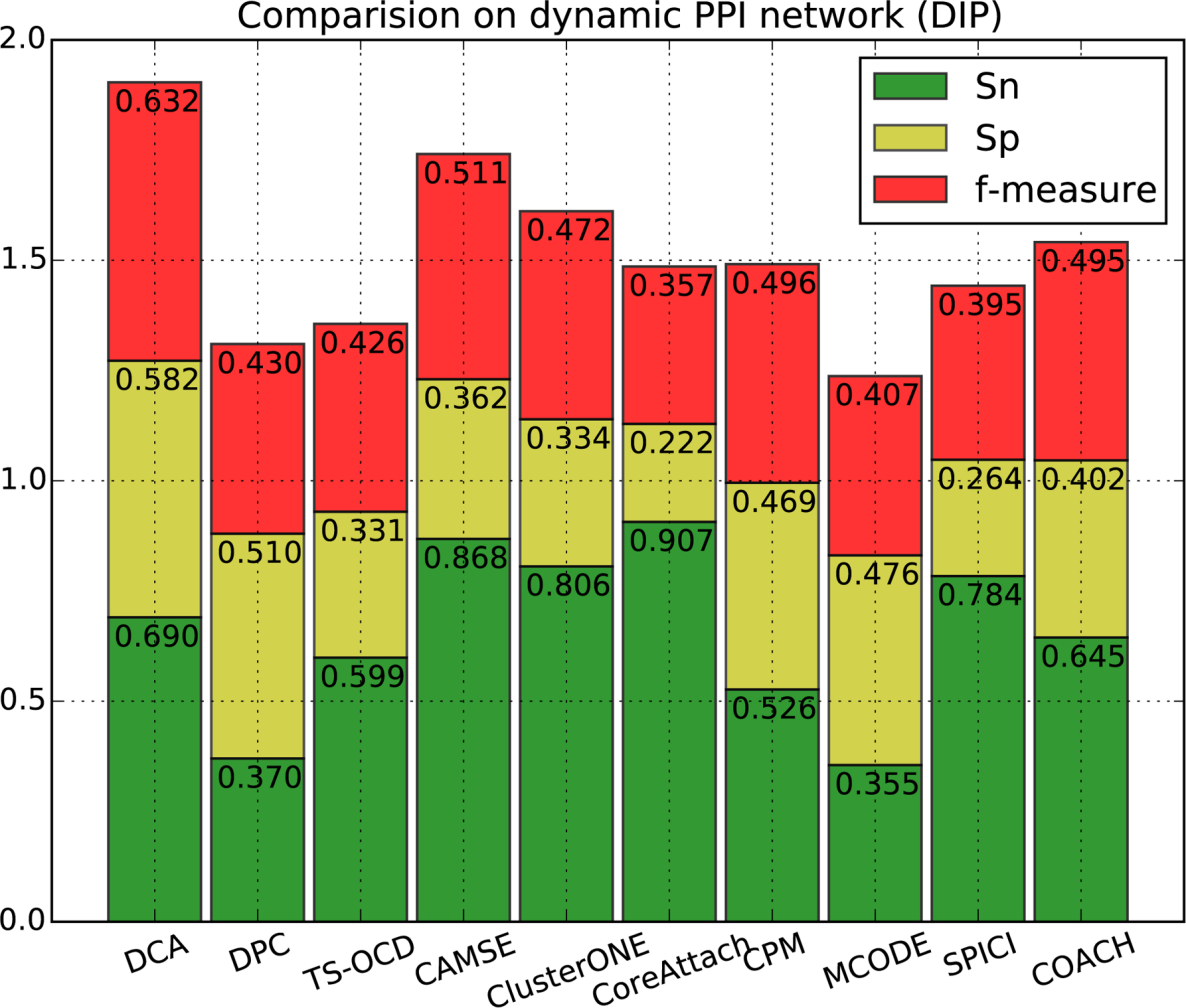
Performance comparison of DCA against other algorithms on dynamic DIP network.

**Fig 4:**
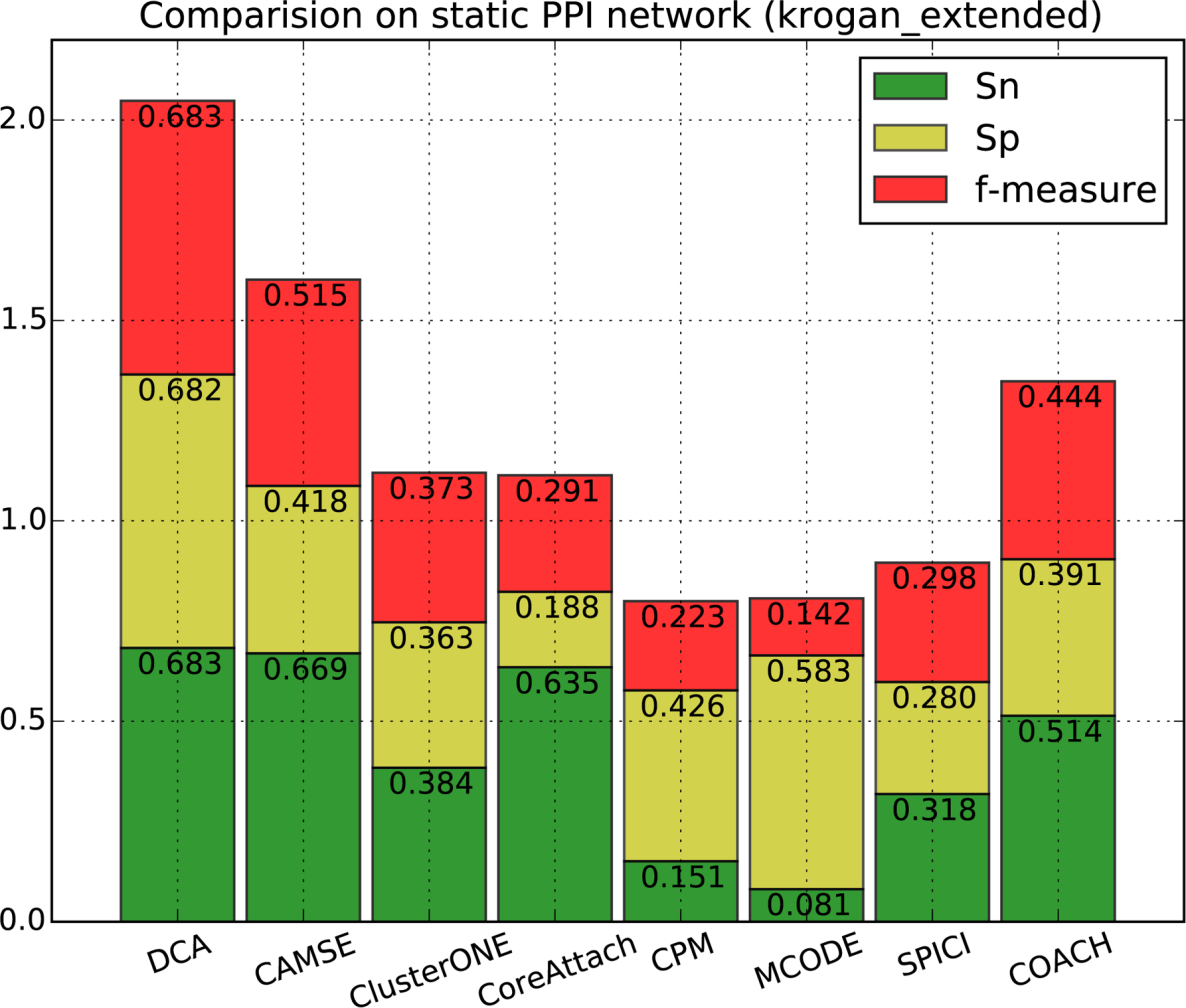
Performance comparison of DCA against other algorithms on static Krogan_extended network.

**Fig 5:**
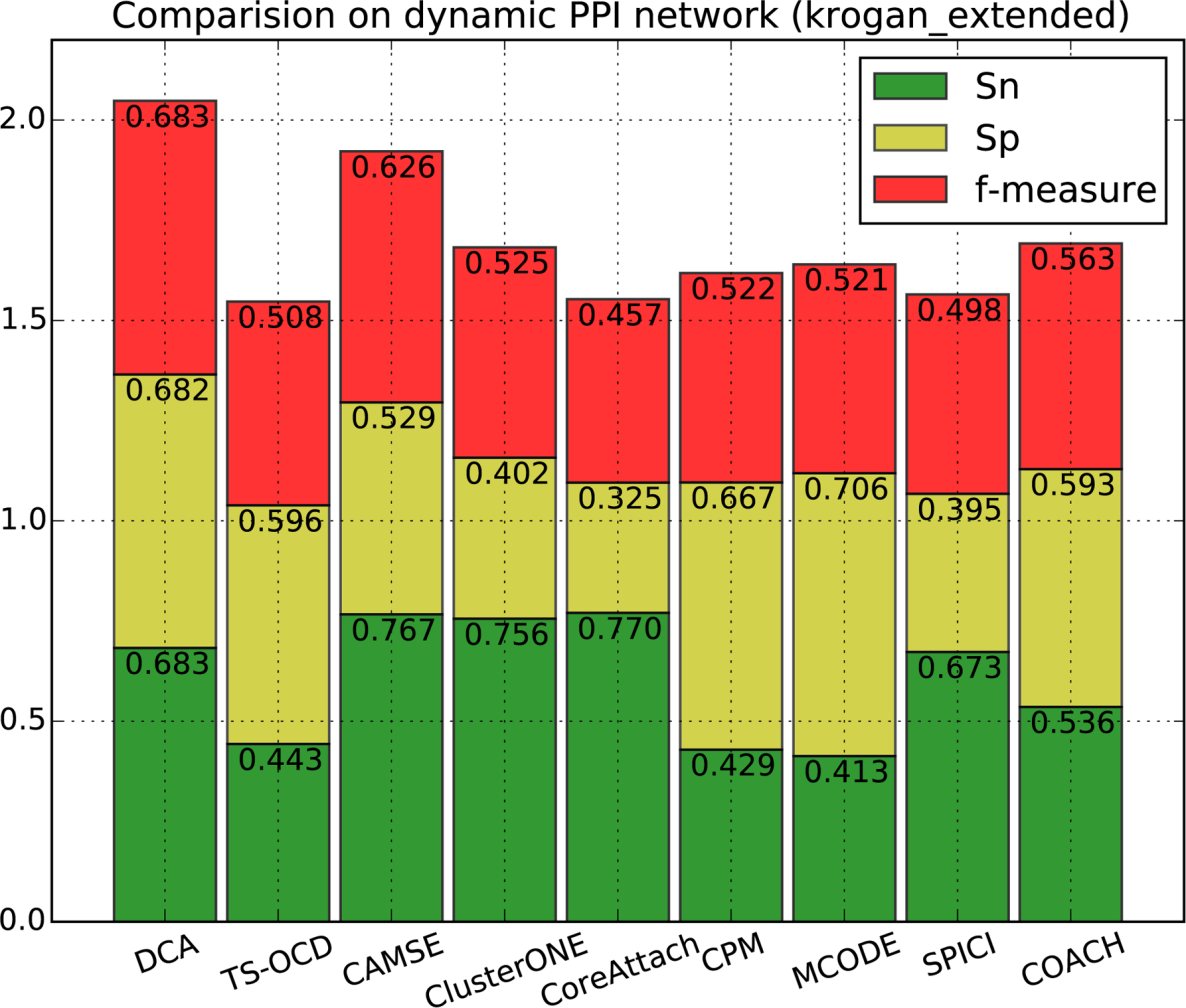
Performance comparison of DCA against other algorithms on dynamic Krogan_extended network.

Fig 2, Fig 3, Fig 4 and Fig 5 show the overall comparison in terms of *Sn*, *Sp* and *F-measure*. On DIP data, the *F-measure* of DCA is 0.632, which is 23.2% higher than the next algorithm CAMSE on static network and 23.7% higher than that on dynamic network. Similarly, on Krogan_extended data, the *F-measure* of DCA is 0.683, which is 32.6% and 17.5% higher than the next algorithm CAMSE on static and dynamic network, respectively. Our DCA method can achieve the highest *F-measure* by providing the highest specificity and comparable sensitivity, which shows that our method can predict protein complexes very accurately.

### P-value analysis

To substantiate the biological significance of predicted protein complexes, we calculate their p-values by the tool, SGD's GO::TermFinder[37]. Table 3 and Table 4 show the distribution of p-value. Using DIP data, 853 out of 885 (96.4%) complexes predicted by DCA are considered to be significant with p-value ≤ 0.01, and it predicts higher proportion of significant complexes than other eight algorithms. Such as in the interval (0, 1e-15], DCA obtains 79(8.9%) complexes while the other algorithms only achieve 4~74(1.1%~4.9%). This result is also consistent with the results on Krogan_extended data where DCA achieves 119(14.6%) significant complexes. Many of our predicted complexes are find to match well with the known complexes. Due to the incompleteness of the benchmark, our non-matched predicted complexes, especially for those with low p-values, may provide potential candidate complexes for biologists to validate.

**Table 3:** Statistical p-values of complexes predicted by DCA algorithm on DIP data.

Algorithms	(0, 1e-15]	(1e-15, 1e-10]	(1e-10,1e-5]	(1e-5, 0.01]	>0.01
DCA	79(8.9%)	107(12.1%)	299(33.8%)	367(41.5%)	32(3.6%)
TS-OCD	22(2.6%)	54(6.4%)	204(24.2%)	510(60.5%)	53(6.3%)
CAMSE	74(3.0%)	125(5.1%)	449(18.5%)	1483(61.0%)	301(12.4%)
ClusterONE	40(2.4%)	73(4.3%)	313(18.6%)	1036(61.3%)	227(13.4%)
CoreAttach	34(1.2%)	45(1.5%)	293(10.0%)	2046(69.7%)	516(17.6%)
CPM	23(4.3%)	42(7.9%)	147(27.7%)	285(53.8%)	33(6.2%)
MCODE	4(1.3%)	32(10.4%)	98(31.7%)	155(50.2%)	20(6.5%)
SPICI	22(1.1%)	49(2.3%)	286(13.7%)	1504(72.0%)	227(10.9%)
COACH	44(4.9%)	58(6.5%)	246(27.4%)	508(56.5%)	43(4.8%)

**Table 4:** Statistical p-values of complexes predicted by DCA algorithm on Krogan_extended data.

Algorithms	(0, 1e-15]	(1e-15, 1e-10]	(1e-10,1e-5]	(1e-5, 0.01]	>0.01
DCA	119(14.6%)	122(14.9%)	272(33.3%)	266(32.6%)	38(4.7%)
TS-OCD	34(10.8%)	23(7.3%)	98(31.2%)	136(43.3%)	23(7.3%)
CAMSE	81(6.8%)	119(10.1%)	252(21.3%)	610(51.5%)	122(10.3%)
ClusterONE	31(2.4%)	50(3.8%)	257(19.6%)	819(62.6%)	151(11.5%)
CoreAttach	29(2.1%)	29(2.1%)	172(12.3%)	945(67.5%)	226(16.1%)
CPM	23(7.7%)	37(12.4%)	92(30.8%)	119(39.8%)	28(9.4%)
MCODE	14(5.2%)	35(12.9%)	95(35.1%)	90(33.2%)	37(13.7%)
SPICI	27(2.7%)	31(3.1%)	186(18.4%)	639(63.3%)	127(12.6%)
COACH	48(10.0%)	45(9.4%)	134(27.9%)	217(45.1%)	37(7.7%)

### HF-measure analysis

**Fig 6:**
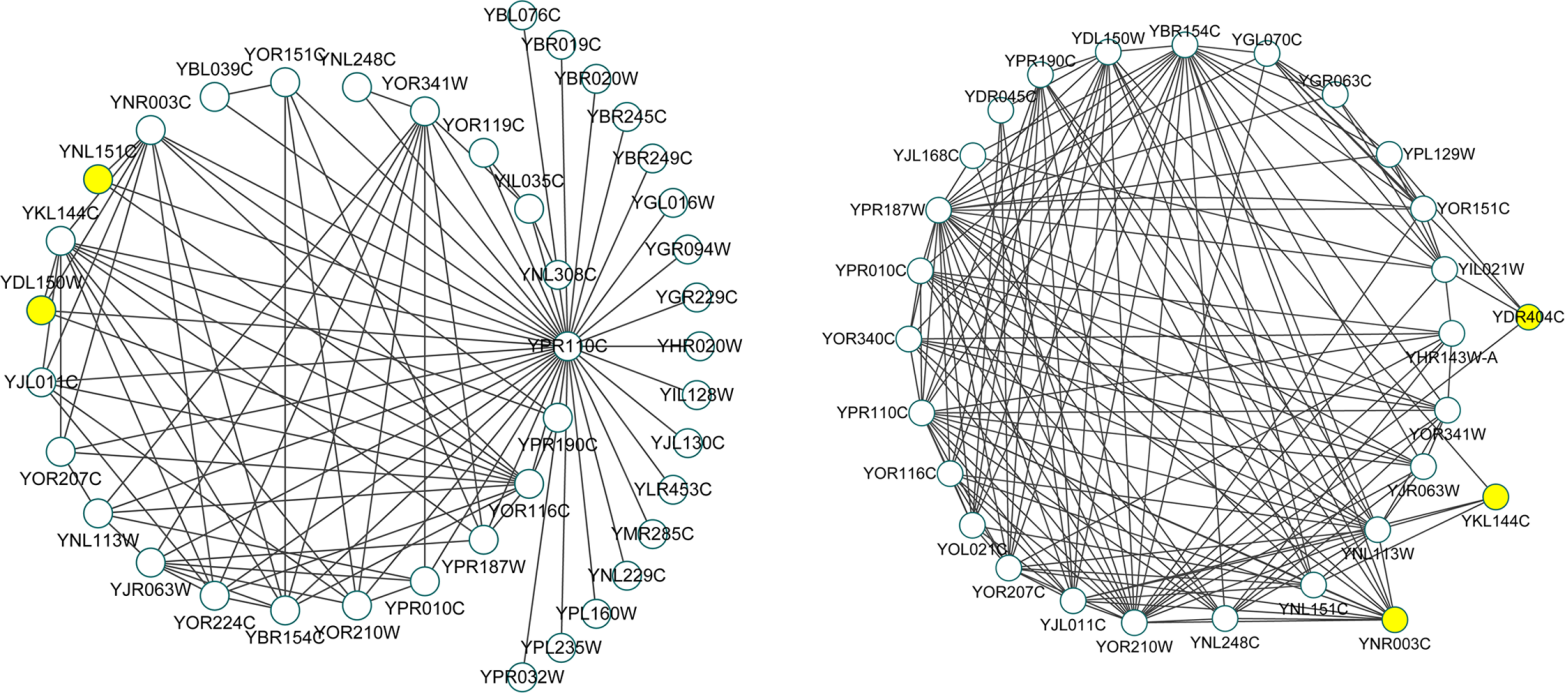
Topology structure illustration of predicted protein complex.

As shown in Table 5, although the hF-measure value of DCA algorithm is little less than TS-OCD, it is 2.1%~12.4% higher than other seven algorithms on DIP dynamic network and 2.8%~14.5% on Krogan_extended dynamic network. Therefore, our DCA algorithm performs significantly better than the most of state-of-the-art techniques. In addition, Table 6 and Table 7 provide ten predicted protein complexes with high hF-measure and low p-value on two dynamic PPI networks. The topology structure of the first complex in the two tables is illustrated in Fig 6, whose GO terms are “tRNA transcription from RNA polymerase III promoter |AmiGO” and “ncRNA transcription | AmiGO”, respectively. From the above analysis we can see that our DCA algorithm detects many useful biological knowledge.

**Table 5:** HF-measure comparison of various algorithms.

	DIP		Krogan_extended	
Algorithms	hF-measure^Tb^	hF-measure^Tf^	hF-measure^Tb^	hF-measure^Tf^
DCA	0.324	0.341	0.350	0.362
TS-OCD	0.333	0.345	0.357	0.363
CAMSE	0.306	0.312	0.330	0.337
ClusterONE	0.314	0.323	0.321	0.328
CoreAttach	0.296	0.303	0.325	0.327
CPM	0.317	0.326	0.341	0.348
MCODE	0.310	0.320	0.311	0.316
SPICI	0.299	0.309	0.319	0.326
COACH	0.316	0.324	0.335	0.342

**Table 6:** Selected protein complexes predicted by DCA algorithm on DIP data.

ID	Core component	Attached component	hfun	hF^Tb^	p-value
11	ypr110c ypr190c ypr187w ypr032w ypr010c ypl235w ypl160w yor341w yor224c yor210w yor207c yor151c yor119c yor116c ynr003c ynl308c ynl248c ynl229c ynl113w ymr285c ylr453c ykl144c yjr063w yjl130c yjl011c yil128w yil035c yhr020w ygr229c ygr094w ygl016w ybr249c ybr245c ybr154c ybr020w ybr019c ybl076c ybl039c	ynl151c ydl150w	GO:0003899	0.521	7.55e-34
55	yor116c ypr190c ypr187w ypr110c yor224c yor207c ynr003c ynl113w ykl144c yjl011c ygl070c yfr037c ydr045c ybr154c	ydl150w ynl151c	GO:0003899	0.612	7.95e-34
39	yol135c yor174w ynr010w ynl189w ymr112c ylr071c ygr104c ygl151w ygl127c yfr019w yfr008w yer022w ydr448w ydr443c ydl005c ybr253w ybr193c	ygl025c	GO:0016455	0.683	3.94e-32
62	yor116c ypr190c ypr187w ypr110c yor207c ynr003c ynl113w ykl144c yjl011c yfr037c ydr045c ybr154c	ykr025w ynl151c ydl150w	GO:0003899	0.583	1.94e-31
65	ygr104c ypr168w ypr070w yor174w yol135c ynr010w ynl236w ylr071c yhr058c yhr041c yer148w yer022w ydr308c ydl005c ybr279w	_	GO:0016455	0.687	6.72e-31
67	ygr104c ypl248c yor174w yol135c ynr010w ynl025c ymr112c ylr071c ygl151w ygl127c yer022w ydl005c ybr253w ybr193c	ygl025c	GO:0016455	0.684	6.72e-31
69	ybr253w ypr168w ypr070w ypl248c yol135c ynl236w ymr112c ygr104c ygl025c yer022w ydr443c ydl005c ybr193c ybr081c	ycr081w	GO:0016455	0.684	6.72e-31
13	ypr110c ypr190c ypr187w ypl235w yor210w yor207c yor151c yor119c yor116c ynr003c ynl308c ynl248c ynl229c ynl113w ymr285c ylr453c ylr238w ylr086w ylr058c ykl144c yjr063w yjl011c yjl008c yil035c yer025w ydl155w ybr245c ybr154c ybr127c ybl076c ybl039c	ynl151c ykr025w ydl150w	GO:0003899	0.507	1.38e-30
53	ydl005c ypr168w ypr070w yor174w yol135c ynr010w ynl236w ylr071c yhr058c yhr041c ygr104c yer157w yer022w ydl153c ycr081w ybr079c	_	GO:0016455	0.667	3.58e-30
58	ybr253w ypl248c yor174w yol135c ynr010w ynl025c ymr112c ylr071c ygr104c ygl151w yer022w ydr448w ydr443c ydl005c ybr193c	ygl025c	GO:0016455	0.667	3.58e-30

**Table 7:** Selected protein complexes predicted by DCA algorithm on Krogan_extended data.

ID	Core component	Attached component	hfun	hF^Tb^	p-value
8	ypr187w yor151c ypl129w yor210w yor116c ynl113w ypr110c ypr010c yor341w yor340c yor207c yol021c ynl248c ynl151c yjr063w yjl168c yjl011c yil021w ygr063c ygl070c ydl150w ybr154c ypr190c yhr143w-a ydr045c	ynr003c ydr404c ykl144c	GO:0003899	0.800	3.96e-51
1	ybr154c yol021c ynl151c ypr086w ypr010c yor207c yjr063w yjl168c yjl011c yfr037c ypr187w ypr133c ypr110c ypl129w yor341w yor340c yor224c yor210w yor151c ynl248c ynl113w ylr200w yil021w ygr063c ygr005c ygl070c ygl043w yfl023w ydr156w ydl150w ydr045c yhr143w-a	ynr003c ykl144c ydr404c	GO:0003899	0.720	7.94e-47
3	yor210w ypr187w ypr190c yor341w ypr110c ypr010c yor340c yor224c yor151c yor116c ynl216w ynl113w ykl054c yhr143w-a ygr005c yer179w yor207c ynl248c yjr063w yjl168c yjl011c yil021w ygl070c yfr037c yer162c ydr156w ydl150w ydl042c ybr279w ybr154c	ynr003c ydr404c	GO:0003899	0.722	2.17e-45
5	ypr110c ypr190c yor116c yfl023w yer162c yel048c ypr187w ynl113w yjr132w yjl076w yfl009w ydl116w ypr010c yor341w yor340c yor210w yor207c yol021c ynl248c ynl186w ynl151c yjr063w yjl011c yhr143w-a ydr156w ydl150w ydl042c ybr154c ydr045c	ynr003c ykl144c	GO:0003899	0.667	4.12e-40
24	ynl113w ypr190c ypr187w ypr010c yor116c ynr003c ynl151c ypr161c ypr110c yor341w yor340c yor210w yor207c ynl248c ykl144c yjr063w yjl011c ydr156w ydr026c ydl150w ydl042c ybr154c	ynr003c ykl144c	GO:0003899	0.704	1.25e-39
10	ypr110c ypr190c yor116c yfl023w yel048c ypr187w ynl113w yjr132w yjl076w yfl009w yor340c yor210w yor207c yol021c ynl248c ynl186w ynl151c ykr025w yjr063w yjl011c yhr143w-a ydr156w ydl150w ybr154c ydr045c	ynr003c ydl042c ykl144c	GO:0003899	0.667	8.83e-39
18	yor210w ypr187w ypr190c ypr110c yor340c yor151c yor116c ynl113w yhr143w-a yor207c ynl248c yml010w ykr025w yjr063w yjl168c yjl011c yil021w yfr037c ydr156w ydl150w ybr279w ybr154c	ynr003c ydl042c	GO:0003899	0.679	3.42e-38
4	ybr154c yol021c ynl151c ypr086w yor207c ykr025w yjr063w yjl168c yjl011c ygr140w yfr037c ypr187w ypr133c ypr110c ypl129w yor340c yor210w yor151c ynl248c ynl113w ylr200w yil021w ygl043w yfl023w ydr156w ydl150w ydr045c ydl042c yhr143w-a	ykl144c ynr003c	GO:0003899	0.635	2.38e-37
20	ypr187w yor151c ypl129w yor210w yor116c ynl113w ypr110c yor340c yor207c yol021c ynl248c ynl151c yjr063w yjl168c yjl011c yil021w ydl150w ybr154c yhr143w-a ydr045c	ydl042c ynr003c	GO:0003899	0.667	1.54e-36
45	yor207c ypr190c ypr187w ypr110c yor224c yor210w yor116c ynl113w yhr143w-a ydr045c ybr154c yol021c ynl151c yjl011c ydr167w ydl150w ydl042c	ykl144c ynr003c	GO:0003899	0.627	1.14e-32

**Fig 7:**
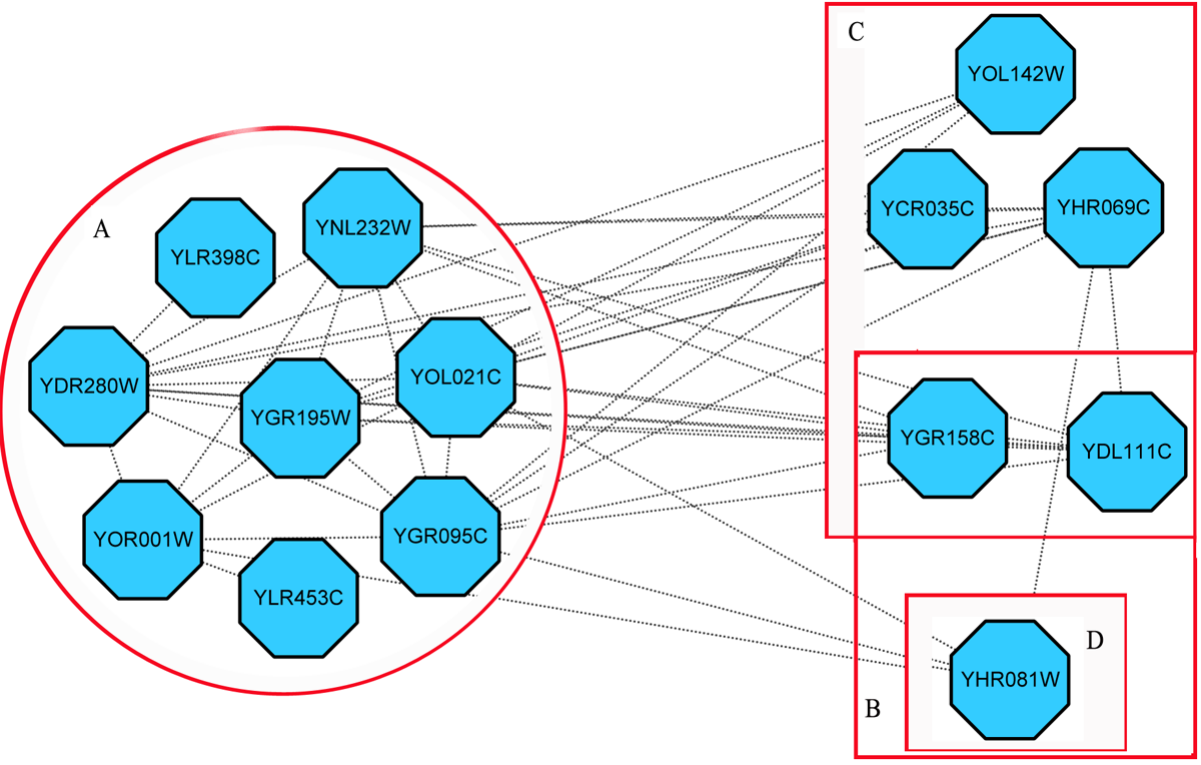
An illustration of predicted protein complex.

Fig 7 illustrates an example of predicted complex, whose core consists of eight proteins in circle *A*. Separately, proteins in blocks *B*, *C* and *D* are the attachments of this complex under different time points. It’s GO annotation is “3'-5'-exoribonuclease activity” (GO:0000175) with p-value 6.93e-20 and hF-measure^*Tb*^ 0.64.

## Conclusions

Protein complexes comprising of multiple highly related proteins are key molecular entities to perform specific cellular functions. The increasing amount of protein-protein interaction data have enabled us to identify protein complexes from PPI networks. However, current computational methods hardly take consideration of both the inherent organization and dynamics within protein complex. This paper presents a new algorithm named DCA for mining protein complex from dynamic PPI network. Its prominent advantage is combing the sequential feature of network with the characteristic of core-attachment structure in complex. The evaluation and analysis of our predictions demonstrate the following advantages of our DCA algorithm over the state-of-the-art completing approaches. First, our new method is fundamentally different from other approaches for its insight into the inherent dynamic organization of protein complexes, which is often neglected in existing algorithms. The consideration of dynamics in cell system made the model simulation more closely to reality. Second, DCA algorithm has achieved significantly higher *F-measure* than existing methods. Thus, our predicted complexes match very well with benchmark complexes. In addition, DCA also performs very well in terms of other metrics such as p-value and hF-measure, indicating that our new algorithm can predict protein complexes very accurately. Our identified complexes, therefore, could be probably the true complexes to help the biologists to get novel biological insights. Although the time sequential gene expression data have much help to explore dynamic protein complexes, many factors need to be considered for deep research on living system, such as living conditions and tissue specifics. The model integrated more dimensional biological data is very important to uncover the mystery of life.
